# Construction of nitrogen critical dilution curve and differences in nitrogen nutrition characteristics in different organs of maize varieties with contrasting nitrogen efficiencies

**DOI:** 10.3389/fpls.2025.1577962

**Published:** 2025-06-18

**Authors:** Shuqin Bao, Xingying Chai, Linzheng Liao, Xiaoying Li, Lin Shi, Xixi Dong, Sida Long, Huifu Li, Hanyu Wang, Zhenghong Li, Mengting Wang, Yun Ren, Qiang Li

**Affiliations:** ^1^ Chongqing Key Laboratory for Germplasm Innovation for Special Aromatic Spice Plants, Institute of Special Plants, College of Landscape Architecture and Life Science, Chongqing University of Arts and Sciences, Chongqing, China; ^2^ College of Biology and Food Engineering, Chongqing Three Gorges University, Chongging, China

**Keywords:** maize, nitrogen efficiency, different organs, nitrogen critical dilution curve, nitrogen nutrition index, nitrogen deficiency

## Abstract

**Introduction:**

This study aimed to establish and verify a critical nitrogen dilution model for different organs of maize varieties with different nitrogen efficiencies and clarify differences in nitrogen nutritional characteristics.

**Methods:**

Two maize varieties (nitrogen-efficient variety ZH 311 and nitrogen-inefficient variety XY 508) were grown under four nitrogen levels to evaluate N dynamics and dry matter accumulation.

**Results:**

The results showed that the critical nitrogen concentration dilution curves based on root dry matter, stem-sheath dry matter, leaf dry matter, and plant dry matter, with coefficient of determination (R²>0.90), all reached significant levels and could be used for nitrogen nutrition diagnosis of maize. However, a and b values of the critical nitrogen concentration models for different organs differed significantly; e.g., the root model had the lowest a value and the leaf model had the lowest b value. ZH 311 exhibited higher a values and lower b values (except in roots) than XY 508. The model established on ZH 311 based on stem-sheath had the highest stability, and the model established on XY 508 based on leaf had the highest stability. Relative yield (RY), nitrogen nutrition index (NNI), and cumulative nitrogen deficit (AND) were significantly correlated at different growth stages of different organs (R²>0.80) using each critical nitrogen concentration dilution curve to predict yield.

**Discussion:**

In the high-nitrogen efficiency variety ZH 311, roots, stem-sheath, leaves, and plants showed weaker responses to AND than those of the low nitrogen-efficiency variety ZH 311 with at all growth stages. ZH 311 roots exhibited stronger responses to NNI at the early growth stage, and stem-sheath, leaves, and plants had stronger responses to NNI at the later growth stage, indicating that the high nitrogen-efficiency variety ZH 311 was not sensitive to nitrogen deficiency and was more efficient for nitrogen nutrition.

## 1 Introduction

The global annual output of maize (*Zea mays* L.) exceeds 12×10^8^ t, accounting for more than 40% of total global food production, making it the largest food crop globally (Sun et al., 2024). High and stable maize yields are crucial for global food security and animal husbandry (Ray et al., 2013). By 2050, global food production must increase by 100% to meet the needs of the global population; however, due to urbanization, farmland degradation, and environmental damage, the arable land area in China is decreasing at a rate of 2.67×10^5^ ha/year, with a decrease of about 30% from 1986 to 2020 (Tilman et al., 2011; Li, 2017; Tu et al., 2024). China is the world ‘s second largest producer of maize; the area of maize planted and its yield in China represent 22% and 24% of the global total, respectively, and it thus makes significant contributions to global maize production (Tian et al., 2020). Therefore, effectively increasing the maize yield per unit area is crucial for ensuring global food security.

Nitrogen application is the simplest and most efficient way to increase crop yield per unit area; however, excessive nitrogen application leads to resource wastage, environmental pollution, and increased production costs (Ma and Diao, 2018). Therefore, diagnosing nitrogen nutrition in crop production and scientific and reasonable nitrogen fertilizer application according to the nitrogen requirement characteristics of different crops at different growth stages have important strategic significance for improving crop yield and nitrogen-use efficiency and realizing high agricultural efficiency, environmental protection, and sustainable development. The critical nitrogen concentration refers to the minimum nitrogen concentration when a crop or certain organ of a crop obtains the maximum biomass during a certain growth period (Li et al., 2015a), and is widely used to assess the nitrogen nutrition status of crops (Zhao et al., 2017; Yue et al., 2016). Tian et al. (2019) established a relationship between the wheat nitrogen nutrition index (NNI) and relative yield (RY) based on a critical nitrogen concentration curve, which can be used for accurate field nitrogen management of wheat. Xue et al. (2006) established a critical post-flowering nitrogen concentration dilution curve model for cotton that can be used to assess post-flowering nitrogen requirements and dynamically monitor nitrogen nutrition to guide timely and accurate fertilization (Wang et al., 2015). Various characteristics (nitrogen-efficiency differences), environmental changes (ecological points and management levels), and model optimization put forward higher requirements for the parameters of the critical nitrogen concentration dilution curve. Therefore, establishing critical nitrogen concentration dilution curves based on dry matter in different organs (rather than traditional plant dry matter) can not only clarify differences in nitrogen nutrition characteristics of various organs but can also help to investigate the physiological function differences of crop nitrogen dynamics resulting from the genotype and environment (Lu et al., 2007).

Breeding and promotion of nitrogen-efficient maize varieties are the most direct and effective means by which to increase maize yield per unit area and reduce nitrogen fertilizer use (Wang et al., 2012). There have been many reports on differences in dry matter accumulation, carbon and nitrogen metabolism, and yield formation of maize varieties with different nitrogen efficiencies; However, studies on differences in critical nitrogen dilution models and nitrogen nutrient characteristics of varieties with different nitrogen efficiency are limited, and current models have limitations, mainly reflected in the failure to consider differences in nitrogen uptake, distribution and metabolism among different maize varieties and their organs. In addition, most models assume a linear relationship between nitrogen concentration and biomass accumulation (Yang et al., 2014). It ignores the complex interactions between nitrogen, carbon and other nutrients. This indicates that the effect of nitrogen efficiency on the critical nitrogen concentration of crops (Nc) model has not been fully explored, and more targeted models need to be developed to accurately predict nitrogen dynamics and optimize nitrogen management, thereby improving crop yield and nitrogen use efficiency (Wang et al., 2019; Liu et al., 2020). Therefore, in this study, maize varieties with different nitrogen efficiencies were used, and critical nitrogen dilution curves were established and verified based on the relationship between the nitrogen concentration and dry matter mass of each organ to clarify differences in the critical nitrogen dilution models of various organs of maize varieties with different nitrogen efficiencies. On this basis, NNI and the cumulative nitrogen deficit (AND) were used to estimate maize yield at key growth stages. A critical nitrogen dilution model was used to calculate the nitrogen production capacity of maize varieties with different nitrogen efficiencies, and differences in their nitrogen nutrient characteristics were identified. This study systematically compared the critical nitrogen dilution models and nitrogen nutrient characteristics of maize varieties with different nitrogen efficiencies for the first time, providing a comprehensive method for diagnosing plant nitrogen status and guidance for precise nitrogen management at each key maize growth stage.

## 2 Materials and methods

### 2.1 Study area and climate

The experiment was conducted at the experimental base of Chongqing University of Arts and Sciences, Wujian Town, Yongchuan District, Chongqing (29°21’N, 105°54’E, altitude 343.5 m), which has a subtropical monsoon climate.

### 2.2 Experimental details

**Figure 1:**
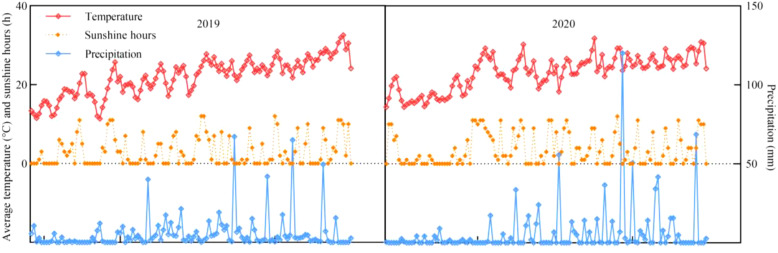
Meteorological factors during maize growth.

The experimental materials were maize varieties with different nitrogen efficiencies selected from previous experiments: nitrogen-efficient variety Zhenghong 311 (ZH 311), and nitrogen-inefficient variety Xianyu 508 (XY 508). These two varieties have similar growth periods in southwestern China (i.e., approximately 120 d). The experiment was conducted during three consecutive maize seasons from April, 2019 to August, 2021 (**Figure 1** shows the weather data of crop growth period). The previous crop was a vegetable, and the soil was purple (Regosols). Basic soil samples were collected from the 0–30 cm soil layer with organic matter content of 16.14 g·kg^-1^, total nitrogen content of 1.63 g kg^-1^, total phosphorus of 0.62 g kg^-1^, total potassium content of 11.55 g·kg^-1^, alkaline hydrolytic nitrogen content of 48.72 mg kg^-1^, available phosphorus content of 2.68 mg kg^-1^, available potassium content of 145.21 mg kg^-1^, and pH of 7.92. The experiment followed a two-factor randomized design with two maize varieties (ZH 311 and XY 508) and four nitrogen fertilizer levels (N1: 0 kg·ha^-1^, N2: 120 kg·ha^-1^, N3: 240 kg·ha^-1^, N4: 360 kg·ha^-1^). Each treatment included 3 replicates, with a total of 24 (8×5 m=40 m^2^) plots, the plant spacing is 25 cm. Maize was planted in wide and narrow rows (1.4 and 0.6 m, respectively) with a density of 52500 plants·ha^-1^. According to the experimental treatment, nitrogen fertilizer was applied in equal amounts before seeding and V12, and phosphate (containing 12.0% P_2_O_5_) and potassium fertilizers (containing 60.0% K_2_O) were used as base fertilizers; application amounts were 600 kg·ha^-1^ of superphosphate and 150 kg ha^-1^ of potassium chloride, respectively. Urea was used as the nitrogen fertilizer (containing 46.4% N).

Maize was sown on March 27, 2019, March 26, 2020, and March 28, 2021, and seedlings were set at the three-leaf stage; maize was harvested on July 29, 2019, July 30, 2020, and July 31, 2021, respectively. Standard field management practices (scheduled irrigation, balanced fertilization, and soil tillage) and integrated pest management strategies (combination of biological control agents and selective pesticides) were implemented in accordance with the Chongqing High-Yield Cultivation Technical Regulations.

### 2.3 Field measurement and index determination

**Figure 2:**
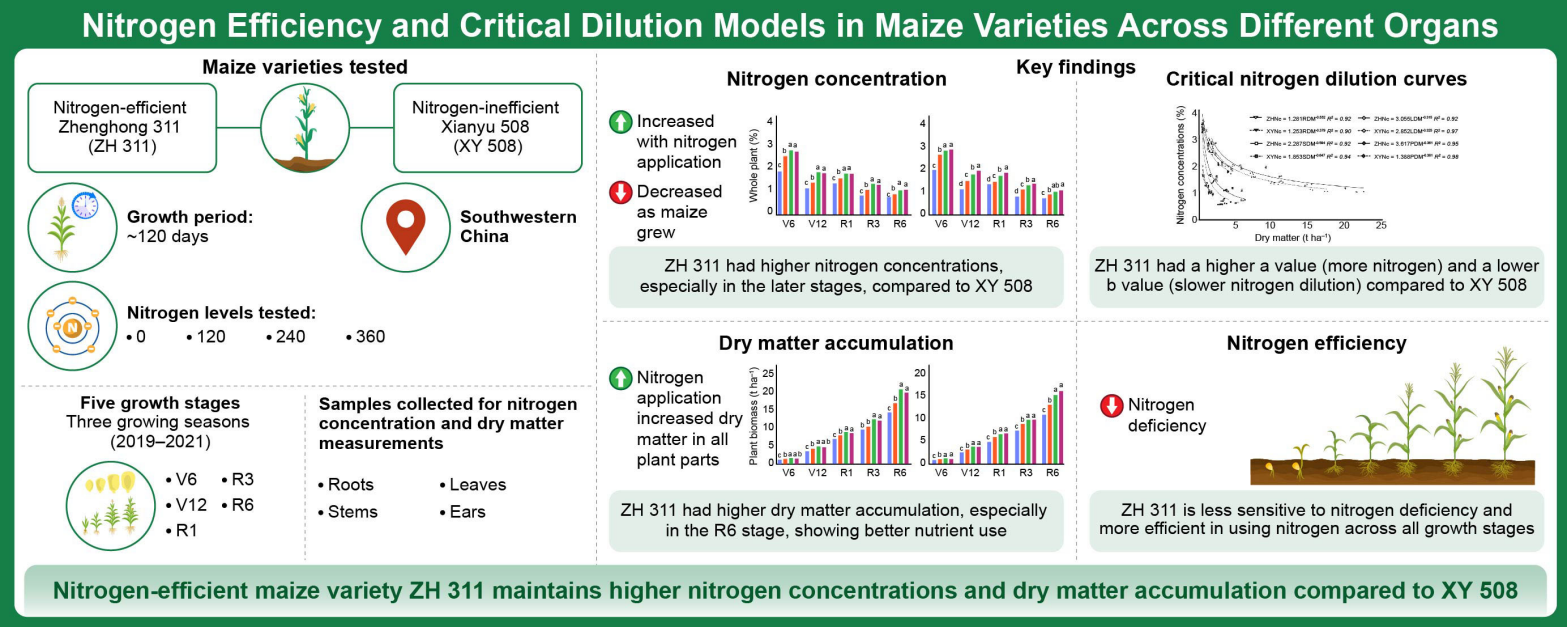
Experiments and technical flowchart of this study.

In the V6, V12, R1, R3, and R6 stages (representing the maize jointing, large horn, silking, filling, and maturity stages, respectively), five representative plants with uniform growth were selected from each plot, and divided into roots (Specifically, a soil column with a length of 0.3 m, width of 0.1 m, and depth of 80 cm was excavated with an iron plate root picker centered on a single plant), stem-sheath, leaves, and ears. Samples were defoliated at 105°C for 30 min, then dried at 80°C to a constant weight and weighed, and dry matter accumulation in roots, stem-sheath, leaves, and individual plants was measured (**Figure 2** shows the experiments and technical flowchart of this study). Samples were then crushed through a 60-mesh sieve and the nitrogen concentration in each organs was determined using the Kjeldahl method with reference to Li et al. (2023). The nitrogen concentration per plant was determined as total nitrogen accumulation per plant/total dry matter accumulation per plant.

At maturity, 30 consecutive ears were harvested from each plot for drying and threshing, and the yield of each treatment was calculated based on a water content of 14%.

### 2.4 Critical nitrogen dilution model

#### 2.4.1 Model construction

The critical nitrogen concentration dilution curve was modeled according to the methodology proposed by Justes et al. (1994). The specific steps applied in this study were as follows: (1) individual plant dry matter (root, stem-sheath, and leaf dry matter) treated with different nitrogen concentrations were divided into nitrogen-limited and non-nitrogen-limited nutrient groups using analysis of variance; (2) linear fitting of dry matter and the nitrogen concentration in the nitrogen-limited nutrient group was carried out, and the dry matter of the non-limited nitrogen nutrient group was averaged and the curve perpendicular to the horizontal axis. The intersection of the two lines corresponded to the critical nitrogen concentration.

Maize critical nitrogen concentration dilution curve model:


(1) $$N_{c}=\text{a}DM^{-b}$$

Here, *N_c_* represents the critical nitrogen concentration of crops; *DM* is the dry matter of each organ (t ha^-1^); parameter a represents the critical nitrogen concentration corresponding to each part of maize when the dry matter of each part is 1 t, and; parameter *b* is the statistical parameter of the slope of the dilution curve of the critical nitrogen concentration.

#### 2.4.2 Model verification

The root mean square error (RMSE) and normalized root mean square error (*n*-RMSE) (Yang et al., 2000) were adopted for model evaluation, These metrics were calculated using Equations 2, 3:


(2) $$\text{RMSE}=\sqrt{\frac{\sum_{i=1}^{n}{\left(Oi-Pi\right)}^{2}}{n}}\;\;$$

(3) $$n-\text{RMSE}=\frac{\text{RMSE}}{\text{S}}\times 100\%$$

where *Oi* and *Pi* are measured and simulated values of the critical nitrogen concentration, respectively; *n* is the sample size, and; S is the average value of the measured data. Model stability was measured according to the standard proposed by Jamieson et al. (1991), i.e., model stability is excellent if *n*-RMSE<10%; model stability is good if 10%<*n*-RMSE<20%; model stability is general if 20%<*n*-RMSE<30%, and; model stability is poor if *n*-RMSE>30%.

### 2.5 Correlation calculations

Referring to the NNI model described by Lemaire et al. (2008), Equation 4 defines the NNI:


(4) $$\text{NNI}=\frac{\text{Na}}{Nc}$$

where NNI is the nitrogen nutrient index; Na is the measured nitrogen concentration, and; *Nc* is the critical nitrogen concentration. Nitrogen nutrition was optimal when NNI=1, excessive when NNI>1, and insufficient when NNI<1. Equation 5 calculates the absolute nitrogen deficit (AND):


(5) $$\text{AND}=N_{\text{cna}}-N_{\text{na}}$$

Here, AND is the nitrogen deficit (kg ha^-1^); *N_cna_* is the crop nitrogen accumulation under the critical nitrogen concentration (kg ha^-1^), and; *N_na_* is actual crop nitrogen accumulation (kg ha^-1^). The nitrogen nutrition status was optimal when AND=0, and nitrogen accumulation was insufficient when AND>0 and excessive when AND<0.

According to the critical nitrogen dilution curve model, when a certain nitrogen concentration in a certain period is determined, the corresponding dry matter accumulation can be estimated and its level can determine the dry matter production capacity under the same nitrogen concentration. It is defined as the nitrogen dry matter production capacity (NDMP, t ha^−1^) and was calculated using Equation 6:


(6) $$NDMP=\sqrt[{b}]{\frac{a}{N\text{t}}}$$

where *N*t is the nitrogen concentration in each organ of the plant, and the *a* and *b* values are the same as those in Equation 1.

### 2.6 Data analysis

Excel 2013 was used for data sorting and analysis, and SPSS (version 26.0) was used for analysis of variance. Variance analysis was performed using the least significant difference for comparisons between treatments at *p*<0.05. Graphpad Prism 9.5 was used for drawing. Field data from 2019 and 2020 were used to build the model, while test data from 2021 were used to verify the model.

## 3 Results and analysis

### 3.1 Changes in nitrogen concentration in different growth periods of maize varieties with different nitrogen efficiencies

**Figure 3:**
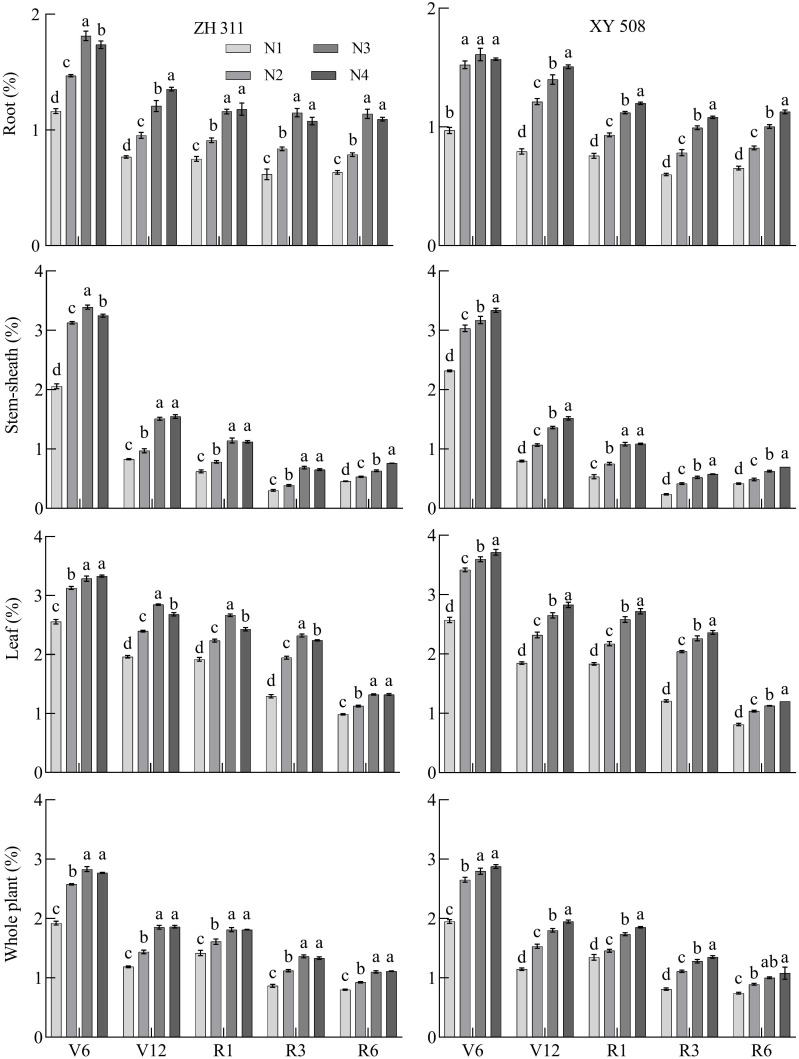
Changes in the nitrogen concentration in maize varieties with contrasting nitrogen efficiencies at different stages (Average data for 2019-2020). ZH 311–Zhenghong 311; XY 508–Xianyu 508; N1–0 kg·ha^-1^; N2–120 kg·ha^-1^; N3–240 kg·ha^-1^; N4–360 kg·ha^-1^; V6–jointing stage; V12–large horn stage; R1–silking stage; R3–filling stage; R6–maturity stage.

Nitrogen concentrations in maize roots, stem-sheath, leaves, and individual plants gradually decreased during growth (**Figure 3**). The root system area decreased from 1.81% in the V6 stage to 0.60% in the R6 stage. Stem-sheath thickness decreased from 3.39% in V6 stage to 0.23% in the R6 stage. The blade area decreased from 3.33% in the V6 stage to 0.81% in the R6 stage, and the rate per plant decreased from 2.83% in the V6 stage to 0.74% in the R6 stage. Nitrogen concentrations in the roots, stem-sheath, leaves, and individual plants of the N-treated maize significantly increased. Compared with N1, the roots of the two varieties increased by 51.92% in the V6 stage, 63.10% in the V12 stage, 43.87% in the R1 stage, 62.19% in the R3 stage, and 54.74% in the R6 stage. The results showed that nitrogen application had the greatest effect on root nitrogen concentrations in maize varieties in the V12 and R3 stages, with increases of>60%. The stem-sheath nitrogen concentration increased by 47.15%, 63.31%, 71.95%, 91.01%, and 42.62%, respectively, indicating that nitrogen application had the greatest effect on the concentration of stem-sheath nitrogen in the R1−R3 stages, with increases>70%. The leaf nitrogen concentration increased by 33.03%, 37.56%, 31.49%, 75.45%, and 32.40%, respectively, indicating that nitrogen application had the greatest effect on leaf nitrogen concentration during the R3 stage; the plant nitrogen concentration increased by 42.05%, 49.14%, 23.89%, 50.29%, and 32.10%, respectively, indicating that nitrogen application had the greatest effect on nitrogen concentration during the R3 stage.

On average, the root nitrogen concentration of ZH 311 (except in the V12 stage) was higher than that of XY 508; those in the V6, R1, R3, and R6 stages were 11.00%, 1.28%, 4.51% and 5.29% higher, respectively. The stem-sheath nitrogen concentration of ZH 311 was higher than that of XY 508 in all stages, and those in the V6, V12, R1, R3, and R6 stages were 1.58%, 3.08%, 5.22%, 15.29%, and 6.17% higher, respectively, than those of XY 508, indicating that ZH 311 maintained a higher stem- sheath nitrogen concentration than XY 508, particularly after the R3 stage. There was no significant difference in leaf nitrogen concentration between the two varieties in the V6-R3 stages, but at R6 stage, ZH 311 was significantly higher than XY 508 (13.94%), indicating that ZH 311 was more effective than XY 508 in maintaining leaf nitrogen concentration and delaying leaf senescence. The nitrogen concentration per plant in ZH 311 was lower than that in XY 508 in the V6 and V12 stages, but higher than that in XY 508 in the R1-R6 stages. The advantage of the nitrogen concentration per plant of ZH 311 was more obvious than that of XY 508 as the growth period was delayed, indicating that the nitrogen concentration per plant of ZH 311 had a certain advantage over XY 508 in the later growth period.

### 3.2 Changes in dry matter of maize varieties with different nitrogen efficiencies in different stages

**Figure 4:**
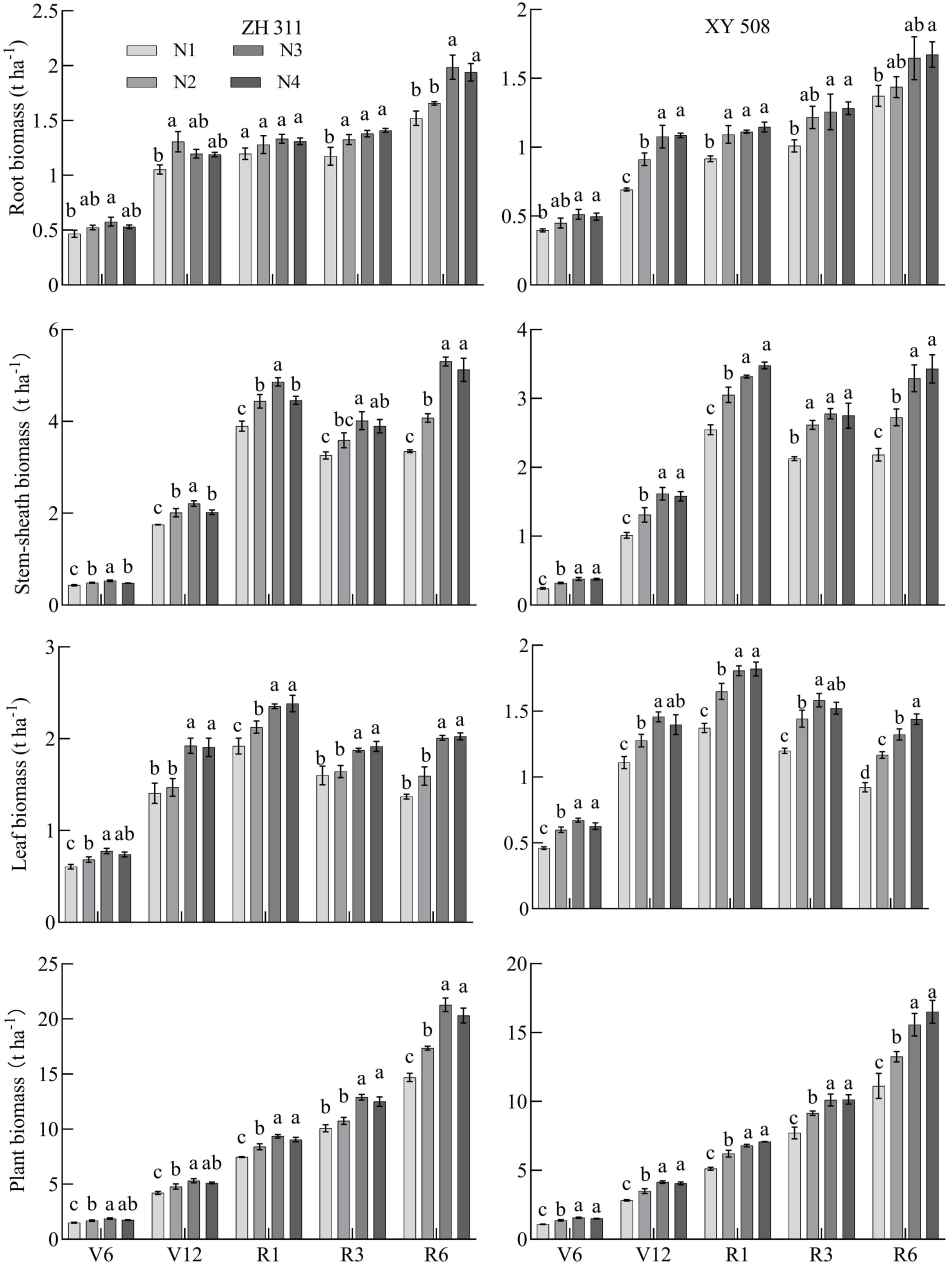
Changes of dry matter accumulation in maize cultivars with contrasting nitrogen efficiencies at different stages (Average data for 2019-2020). ZH 311–Zhenghong 311; XY 508–Xianyu 508; N1–0 kg·ha^-1^; N2–120 kg·ha^-1^; N3–240 kg·ha^-1^; N4–360 kg·ha^-1^; V6–jointing stage; V12–large horn stage; R1–silking stage; R3–filling stage; R6–maturity stage.

Dry matter of maize root, stem-sheath, leaf, and individual plant increased gradually with growth, and the dry matter of maize root increased from 0.39 t ha^-1^ in the V6 stage to 1.98 t ha^-1^ in the R6 stage. Stem-sheath dry matter increased from 0.24 t ha^-1^ in the V6 stage to 5.30 t ha^-1^ in the R6 stage. Blade dry matter increased from 0.46 t ha^-1^ in the V6 stage to 2.38 t ha^-1^ in the R6 stage. Single plant dry matter increased from 1.09 t ha^-1^ in the V6 stage to 21.29 t ha^-1^ in the R6 stage (**Figure 4**). Roots, stem-sheath, leaves, and dry matter per plant in nitrogen-treated maize increased gradually. Compared with N1, the roots of the two varieties were 19.30% higher (*p*<0.05) in the V6 stage, 29.19% higher in the V12 stage, 14.77% higher in the R1 stage, 20.20% higher in the R3 stage, and 19.12% higher in the R6 stage. Stem-sheaths were 26.60% higher in the V6 stage, 29.59% higher in the V12 stage, 22.17% higher in the R1 stage, 21.59% higher in the R3 stage, and 44.25% higher in the R6 stage. The blades were 28.12% higher in the V6 stage, 24.90% higher in the V12 stage, 22.87% higher in the R1 stage, 18.88% higher in the R3 stage, and 38.95% higher in the R6 stage. Plants increased by 25.21% in the V6 stage, 27.68% in the V12 stage, 24.25% in the R1 stage, 22.78% in the R3 stage, and 34.58% in the R6 stage. Nitrogen application significantly promoted dry matter accumulation at different maize growth stages, and increases in the dry matter of the stem-sheath and whole plant were particularly significant in the R6 stage. These results indicated that appropriate nitrogen application could not only promote growth of each maize organ but also contribute to increasing dry matter with advancement of the growth cycle.

The average dry matter performance of the ZH 311 root system was significantly higher than that of XY 508 during the V6, V12, R1, R3, and R6 stages, during which the root dry matter quality was 12.22%, 16.09%, 18.70%, 10.63%, and 17.835 higher, respectively. ZH 311 roots had a stronger soil nutrient absorption capacity than XY 508 roots during the critical growth period. Similarly, the performance of ZH 311 in the stem-sheath and leaf was higher than that of XY 508 at all stages, particularly in the R6 stage; stem-sheath dry matter was 53.11% higher and leaf dry matter was 44.37% higher, indicating that ZH 311 had considerable advantages in nutrient absorption and resource utilization efficiency in the later growth period. The dry matter per plant of ZH 311 was higher than that of XY 508 from stages V6-R6, particularly in the R1 stage (35.41% higher), reflecting the comprehensive performance advantages of ZH 311 in late growth, particularly in terms of dry matter accumulation and nitrogen utilization, which have potential application value in improving crop yield and quality.

### 3.3 Construction of nitrogen critical dilution curve in different maize organs

**Figure 5:**
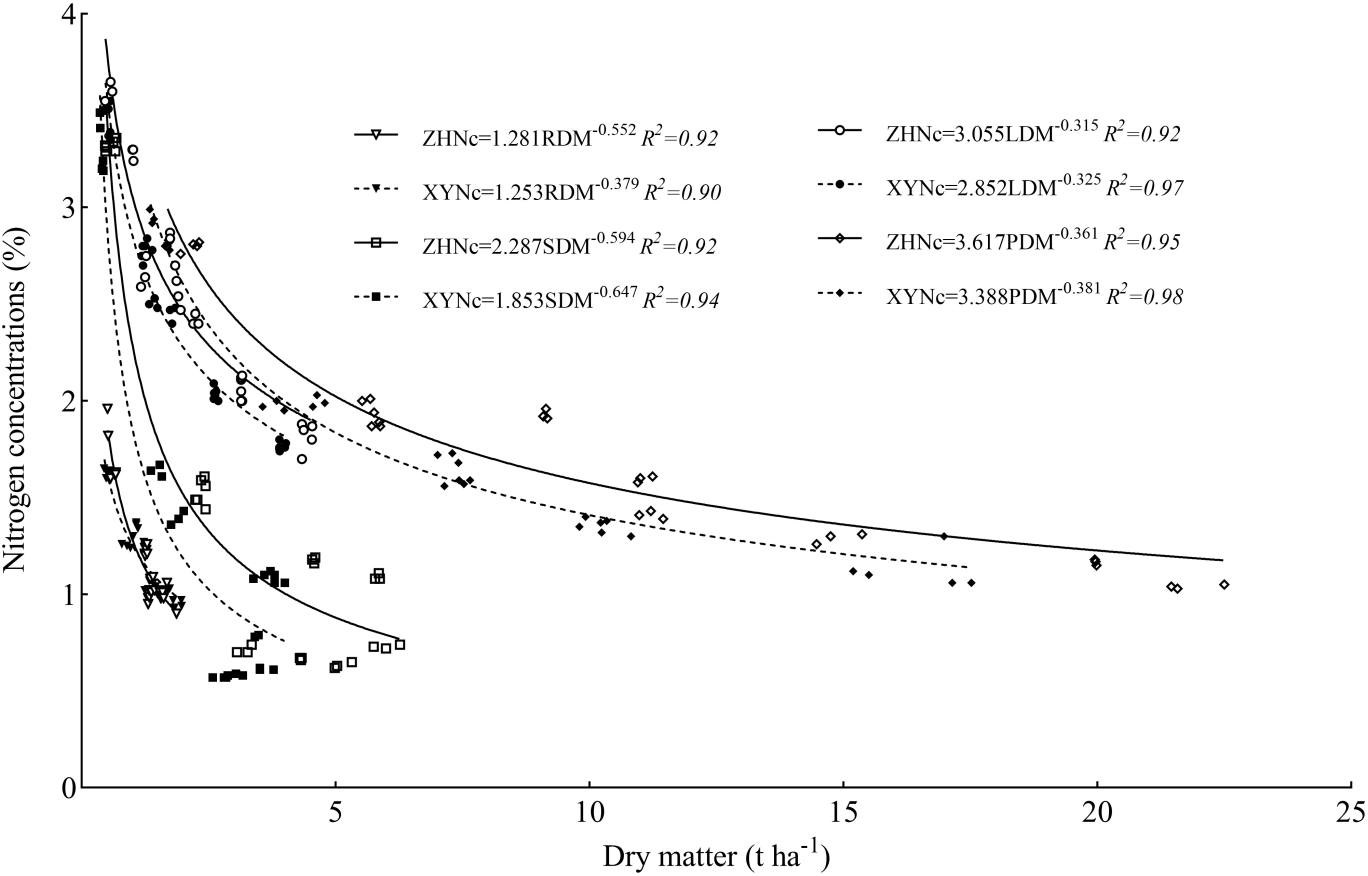
Nitrogen critical dilution curves in different maize organs.

The critical nitrogen concentration dilution curve (**Figure 5**) was established based on the relationship between the dry matter and nitrogen concentration of maize roots (RDM), stem-sheath (SDM), leaves (LDM), and plants (PDM); *R* ^2^ values of the critical nitrogen concentration dilution curve of each organ of the two varieties were>0.90. The critical nitrogen dilution curve parameters of the different maize organs differed significantly, among which plant dry matter had the highest a value (3.617 and 3.388), the root a value was the lowest (1.281 and 1.253), the stem-sheath a value was the highest (0.594 and 0.647), and the leaf a value was the lowest (0.315 and 0.325). There were also significant differences in critical nitrogen dilution curve parameters between the two maize varieties. The critical nitrogen concentration dilution curves for ZH 311 roots, stem-sheath, leaves, and plants (1.281, 2.287, 3.055, and 3.617, respectively) were higher than those for XY 508 (1.253, 1.853, 2.852, and 3.388, respectively). The critical nitrogen concentration dilution curve of the ZH 311 root system (0.552) was higher than that of the XY 508 root system (0.379), and those of the stem-sheath (0.594), leaf (0.315), and plant (0.361) were lower than those of XY 508 (0.647, 0.325, and 0.381, respectively).

### 3.4 Verification of critical nitrogen concentration dilution curve

**Figure 6:**
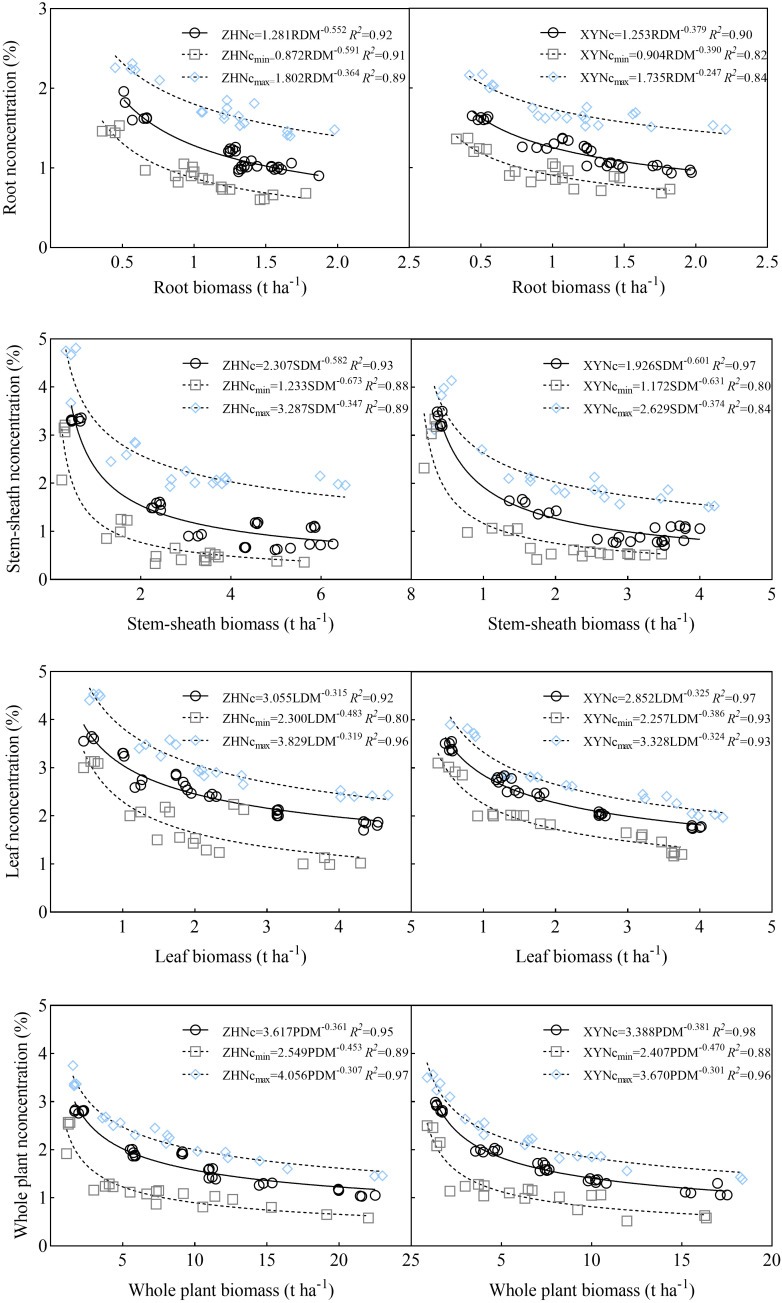
Critical nitrogen dilution curve validation.

The critical nitrogen concentration curve was verified using 2021 data. The results showed that the critical nitrogen concentration dilution curve could divide maize into growth conditions with and without nitrogen restriction. Under nitrogen restriction, all data points fell below the *Nc* dilution curve; whereas, without nitrogen restriction, all data points fell on or above the *Nc* dilution curve (**Figure 6**). Simultaneously, to verify the model accuracy, the measured dry matter data points were introduced into Equation 1 to calculate the simulated value of the critical nitrogen content, which was compared with the observed value (**Table 1**). Deviations based on the root and plant models of the two varieties were 10.85% and 9.50%, and 16.14% and 14.94%, respectively, and the stability of the<20% model was high; deviations based on the stem-sheath and leaf models were 6.77% and 8.64%, and 7.70%, and 5.15%, respectively, and the stability of the<10% model was excellent. Therefore, the critical nitrogen dilution curve model for different maize organs constructed in this study had high accuracy, indicating that the critical nitrogen concentration dilution curve for different organs of maize varieties with different nitrogen efficiencies constructed in this study can be used for nitrogen nutrition diagnosis of maize.

**Table 1:** Observed and simulated values of critical nitrogen concentrations in maize.

Stage	Root				Stem-sheath				Leaf				Plant			
	ZH 311		XY 508		ZH 311		XY 508		ZH 311		XY 508		ZH 311		XY 508	
	OV	SV	OV	SV	OV	SV	OV	SV	OV	SV	OV	SV	OV	SV	OV	SV
V6	1.55	1.37	1.42	1.32	2.95	2.58	2.97	2.51	3.02	3.19	3.06	3.01	2.53	2.03	2.57	2.00
V12	1.07	1.34	1.23	1.29	1.22	1.28	1.19	1.26	2.13	1.68	2.05	1.88	1.64	1.11	1.55	1.12
R1	1.02	1.27	1.05	1.24	0.92	0.95	0.86	0.90	1.65	1.33	1.67	1.37	1.63	0.88	1.62	0.93
R3	0.92	1.23	0.88	1.22	0.51	0.52	0.44	0.48	1.51	1.23	1.51	1.25	1.18	0.76	1.13	0.83
R6	0.88	1.01	0.84	1.06	0.60	0.61	0.56	0.57	1.23	1.08	1.12	1.14	0.98	0.74	0.96	0.80
RMSE	0.118		0.103		0.084		0.104		0.147		0.097		0.257		0.234	
*n*-RMSE	10.85%		9.50%		6.77%		8.64%		7.70%		5.15%		16.14%		14.94%	

OV, Observed value; SV, Simulated value.

### 3.5 Relationship between NNI, AND, and RY

**Figure 7:**
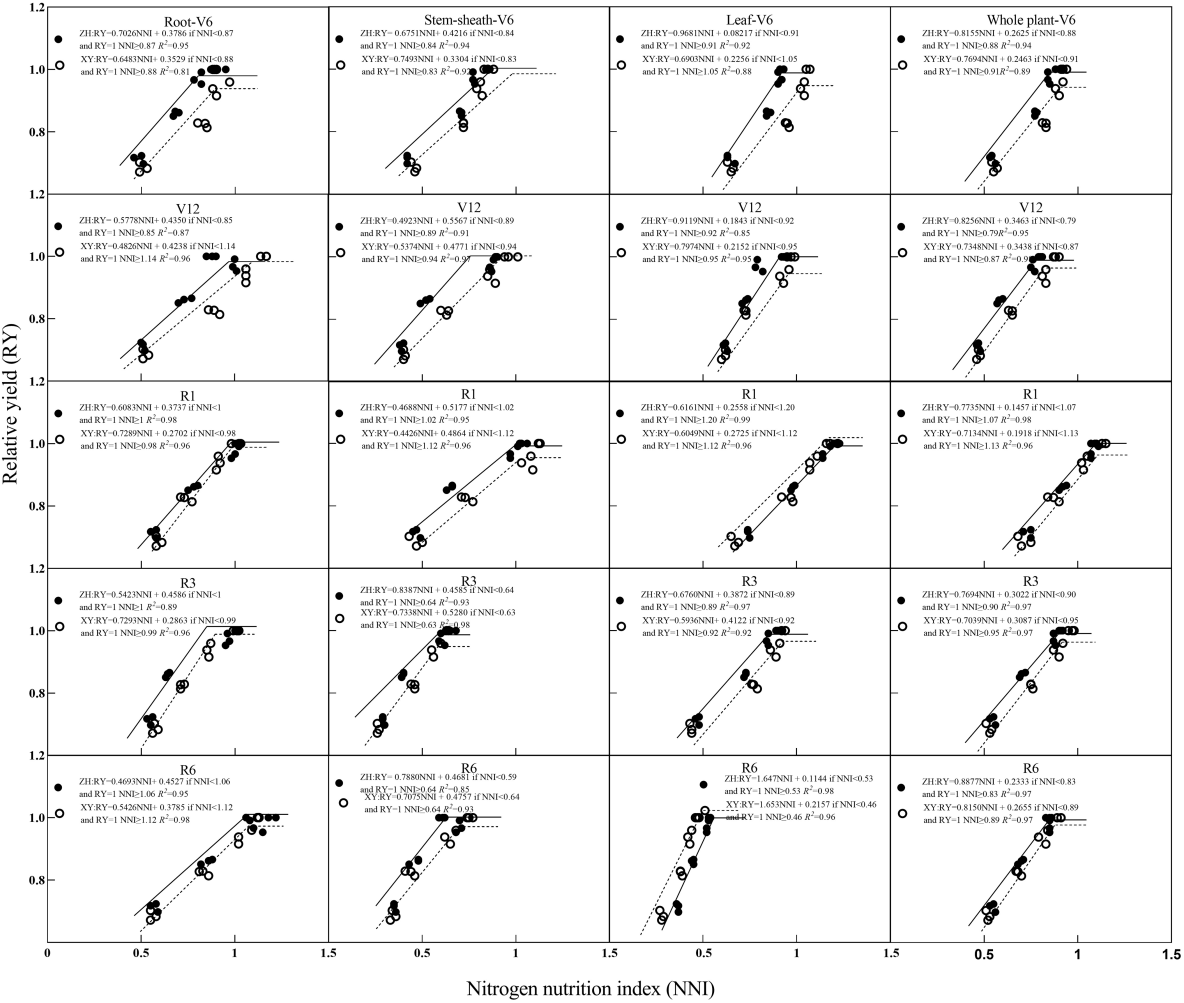
Relationships between relative yield (RY) and nitrogen nutrition index (NNI) obtained from critical nitrogen concentration curves. V6–jointing stage; V12–large horn stage; R1–silking stage; R3–filling stage; R6–maturity stage.

Relationships between NNI and RY and between RY are shown in **Figures 6** and **7**. With an increase in NNI, RY exhibited a linear growth trend until it no longer increased with an increase in NNI, and its change trend presented a linear and stable mode. With an increase in AND, RY showed a trend of initially remaining constant and then decreasing linearly. Based on the critical nitrogen concentration dilution curve of root dry matter, the *R* ^2^ value between NNI, AND, and RY ranged from 0.815–0.984 and between AND and RY ranged from 0.818–0.988. Based on the critical nitrogen concentration dilution curve of the stem-sheath dry matter, the *R* ^2^ value between NNI and RY ranged from 0.852–0.985 and that between AND and RY ranged from 0.757–0.987. Based on the critical nitrogen concentration dilution curve of dry matter, the *R* ^2^ value between NNI and RY ranged from 0.853–0.994 and between AND and RY ranged from 0.735–0.991. Based on the critical nitrogen concentration dilution curve of plant dry matter, the *R* ^2^ value between NNI and RY ranged from 0.894–0.983 and that between AND and RY ranged from 0.890–0.984. Relationships among RY, NNI, and AND were determined based on the critical nitrogen concentration curves established for maize roots, stem-sheath, leaves, and plant dry matter. *R* ^2^ values of each organ of the two varieties were all > 0.70 in each stage; therefore, the effect on yield could be evaluated according to the nitrogen nutrient state (NNI and AND) of crop vegetative growth.

Further analysis showed that with an increase in NNI, maize RY first increased and then stabilized (**Figure 7**). At most growth stages, the RY of ZH 311 was higher than that of XY 508, and the curve of ZH 311 was generally steeper and had a higher slope than that of XY 508 (**Figure 7**), indicating that ZH 311 was able to use nitrogen more efficiently and convert it into biomass. The root difference between ZH 311 and XY 508 was greatest during the R1 stage, with ZH 311 showing higher RYs at an NNI of 1, suggesting that ZH 311 roots may be more efficient at absorbing nutrients when the nitrogen supply is adequate. The largest difference in stem-sheaths was during the R6 stage, when ZH 311 showed higher RYs at an NNI of 0.59, suggesting that ZH 311 stem-sheaths were more efficient in nutrient transport in the absence of nitrogen. The NNI yields of ZH 311 at different stages were higher than those of XY 508, and ranged from 0–1.5, indicating that ZH 311 leaves could photosynthesize more efficiently under deficient or sufficient nitrogen conditions. The largest difference in plant parts was observed between the V6 and V12 stages, and ZH 311 showed a higher RY at an NNI of 0.79, suggesting that whole ZH 311 plants could grow more efficiently in the absence of nitrogen. Overall, the yield advantage of ZH 311 over that of XY 508 was particularly significant, with higher nitrogen-use efficiency in R1 and R3 stages, better grain filling and ripening, and ultimately higher yields.

**Figure 8:**
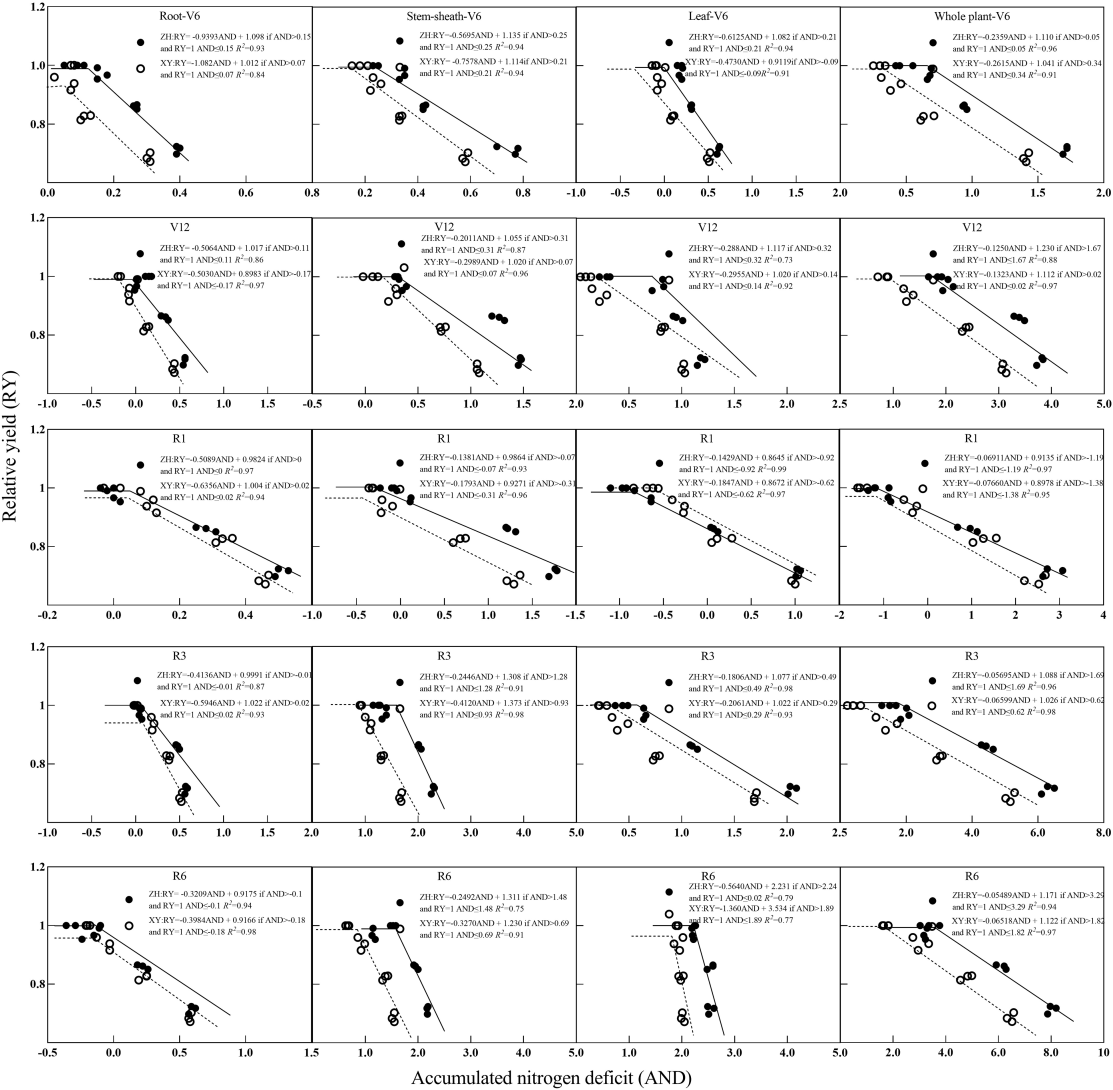
Relationships between relative yield (RY) and accumulated nitrogen deficit (AND) obtained from critical nitrogen concentration curves. V6–jointing stage; V12–large horn stage; R1–silking stage; R3–filling stage; R6–maturity stage.

Differences were observed between ZH 311 and XY 508 in the root, stem-sheath, leaf, and plant yields depending on the crop growth stage (**Figure 8**). In roots and stem-sheaths, ZH 311 showed a high yield in both the V6 and V12 stages. This showed that ZH 311 had higher nitrogen-use efficiency and was able to use limited nitrogen resources more effectively to promote growth and development of organs such as roots and stem-sheaths. In leaves, ZH 311 and XY 508 showed the largest differences in yield during the R1 stage, and ZH 311 showed a smaller yield decline and higher *R*² value, indicating that it was better adapted to nitrogen deficiency and could reduce the effect on reproductive organ development through more efficient nitrogen use or distribution mechanisms. ZH 311 showed high yields in both the V6 and V12 stages, with the highest yield observed in the R6 stage. In summary, ZH 311 was more adaptable to nitrogen deficiency throughout the growth cycle, particularly during the reproductive growth phase; whereas, XY 508 showed high sensitivity to nitrogen deficiency during the early vegetative growth phase, which may affect its overall productivity. These results help elucidate the differences in nitrogen use and yield between the two varieties, as well as their performance at different growth stages and in different organs.

### 3.6 Differences in nitrogen dry matter production capacity of maize varieties with different nitrogen-use efficiencies

Nitrogen concentrations in maize roots, stem-sheaths, leaves, and individual plants gradually decreased with growth (**Figure 3**); whereas, dry matter gradually increased with growth (**Figure 4**). With a decrease in the nitrogen concentration, dry matter accumulation in maize roots, stem-sheaths, leaves, and individual plants increased significantly; however, there were significant differences among the varieties (**Table 2**). In ZH 311, root (except in R3 and R6 stages), stem-sheath, leaf, and single plant NDMP were significantly higher than those of XY 508, and NDMP was 55.17%, 33.33%, 13.33%, -6.38%, and -28.13% higher in V6, V12, R1, R3 and R6 stages, respectively; stem-sheath NDMP were 32.43%, 34.04%, 36.51%, 40.45%, and 46.04% higher, respectively; leaf NDMP were 22.64%, 23.26%, 26.0%, 32.22%, and 32.55% higher, respectively. Plant NDMP was 21.74%, 25.23%, 29.32%, 38.87%, and 43.13% higher, respectively, indicating that the nitrogen-efficient variety ZH 311 had stronger dry matter production capacity than the nitrogen-efficient variety XY 508 at the same nitrogen concentration, and that the differences in nitrogen dry matter production capacity of stem-sheath, leaves, and individual plants increased with decreases in the nitrogen concentration.

**Table 2:** Differences in nitrogen dry matter production capacity (NDMP) of maize varieties with contrast nitrogen efficiency.

Different stage	Nitrogen content (g kg^-1^)	Root (t ha^-1^)		Nitrogen content (g kg^-1^)	Stem-sheath (t ha^-1^)		Nitrogen content (g kg^-1^)	Leaf (t ha^-1^)		Nitrogen content (g kg^-1^)	Plant (t ha^-1^)	
		ZH	XY		ZH	XY		ZH	XY		ZH	XY
V6	2.0	0.45a	0.29b	3.5	0.49a	0.37b	3.5	0.65a	0.53b	3.0	1.68a	1.38b
V12	1.7	0.60a	0.45b	3.0	0.63a	0.47b	3.0	1.06a	0.86b	2.5	2.78a	2.22b
R1	1.4	0.85a	0.75b	2.5	0.86a	0.63b	2.5	1.89a	1.50b	2.0	5.16a	3.99b
R3	1.1	1.32b	1.41a	2.0	1.25a	0.89b	2.0	3.84a	2.98b	1.5	11.45a	8.49b
R6	0.8	2.35b	3.27a	1.5	2.03a	1.39b	1.5	9.57a	7.22b	1.0	35.21a	24.60b
	Mean	1.11 b	1.23a		1.05a	0.75b		3.40a	2.62b		11.26a	8.23b

## 4 Discussion

### 4.1 Establishment of critical nitrogen concentration dilution curves for different maize organs

Nitrogen nutrition diagnosis and nitrogen nutrition status of crops can be determined by establishing a crop critical nitrogen concentration dilution curve (Cao et al., 2020; Li et al., 2015b). Previous studies have established and verified a series of critical nitrogen concentration dilution curves based on leaf (Jia and Fu, 2020), stem-sheath (Lu et al., 2019), ear (Li et al., 2015a), and leaf areas (Lu et al., 2021) of plants. However, because of the differences in nitrogen absorption, distribution, and utilization of various plant indices, models of critical nitrogen dilution curves for various organs differed significantly (An et al., 2019). In this experiment, the determination coefficients (*R* ^2^>0.90) of the critical nitrogen concentration dilution curves established based on RDM, SDM, LDM, and PDM all reached significant levels and had higher inter-year stability, which could be used for diagnosing maize nitrogen nutrition in Southwest China. Values of parameters a and b of the critical nitrogen concentration models based on RDM, SDM, LDM, and PDM differed significantly, among which the a value of the root model was the smallest, which is consistent with the results of Lan et al. (2022). Because the root biomass was significantly lower than that of other organs, and its nitrogen concentration in the early growth stage was also significantly lower than that of other organs, the dilution effect of nitrogen concentration was lower and sensitivity was higher. The b value based on the leaf model was the lowest, which was consistent with the results of Su et al. (2021) and Ata et al. (2017). Because leaves are the most important metabolic organs of plants, a large amount of nitrogen is transferred from plant roots and stem-sheath to leaves during growth to maintain their high nitrogen concentrations and ensure efficient operation of photosynthesis; therefore, the reduction in the leaf nitrogen concentration was much lower than that of roots and stem-sheaths (Lu et al., 2024). The results of this experiment also showed that the nitrogen concentration in maize leaves at each stage was significantly higher than that in the roots and stem-sheaths, further confirming the above conclusion.

Further analysis showed that the a value of the critical nitrogen concentration dilution model for all organs of the nitrogen-efficient variety ZH 311 was higher than that of the nitrogen-inefficient variety XY 508, which was inconsistent with the results of An et al (An et al., 2019; Zhao et al., 2022), mainly because the nitrogen-efficient variety ZH 311 selected in this experiment was a nitrogen-efficient absorption type. The nitrogen concentration per unit biomass (parameter a) of ZH 311 was higher than that of XY 508, which was beneficial for maintaining a higher nitrogen concentration and delaying aging of various organs during later growth periods. Previous studies have found that ZH 311 has advantages over XY 508 in dry matter production, nitrogen accumulation, and yield, mainly because of the later growth period (Li et al., 2016a, Li et al., 2016b), which also supports this conclusion. Furthermore, the b value of the critical nitrogen concentration dilution model for all organs of the nitrogen-efficient variety ZH 311 (except roots) was lower than that of the nitrogen-inefficient variety XY 508, which is consistent with the results of Du et al. (2020). The nitrogen-efficient variety was able to absorb and utilize nitrogen more effectively, thereby slowing the nitrogen dilution rate and increasing its stem, leaf, and plant nitrogen concentrations compared to those of the nitrogen-inefficient variety. This is particularly consistent with the results of this experiment during the later growth period (Li et al., 2022). The root b value of the high nitrogen efficiency varieties was higher than that of the low nitrogen efficiency varieties, which was conducive to maintaining a higher nitrogen concentration in the early growth stage, improving nitrogen accumulation, and promoting morphogenesis; whereas, the nitrogen concentration decreased rapidly in the later growth stage, improving nitrogen transport and utilization. Therefore, the root nitrogen concentration of ZH 311 in the early growth stage was significantly higher than that of XY 508 in later growth stages. Consistent with the results of Du et al. (2020), this study demonstrated efficient nitrogen use in nitrogen-efficient varieties.

In addition, the *n*-RMSE evaluation model was used to prove that the nitrogen-efficient variety ZH 311 had the highest stability based on the stem-sheath (*n*-RMSE=6.77%), which was consistent with the results of Su et al. (2021) This was mainly because the difference in nitrogen efficiency was mainly caused by the difference in stems rather than those in leaves. This reflected the high nitrogen dilution rate (i.e., the highest b value) of the maize stem-sheath, which, on the one hand, is related to rapid dry matter accumulation in the stem-sheath of nitrogen-efficient varieties; on the other hand, it reflected the efficient post-flowering nitrogen transport of nitrogen-efficient varieties to the ear to maintain rapid grain growth, which is consistent with the higher nitrogen transport efficiencies of nitrogen-efficient maize varieties than those of nitrogen-inefficient varieties. The model stability of the nitrogen-inefficient variety XY 508 based on leaves was the highest (*n*-RMSE=5.15%), which is consistent with the results of Fu et al. (2020), where the low nitrogen-efficiency variety XY 508 had the highest leaf a value, highest demand for nitrogen concentration per unit biomass, lowest leaf b value, and lowest nitrogen dilution rate. Nitrogen was preferentially transferred to the leaves during the later growth period to maintain the nitrogen concentration during this period. Therefore, leaf senescence during the late growth period is key to limiting yield in nitrogen-inefficient varieties (Kosgey et al., 2013; Mu et al., 2015).

### 4.2 Differences in nitrogen nutrition characteristics in different maize organs

A relationship model between crop NNI, AND, and RY can be constructed to effectively evaluate the nitrogen nutrition status of crops and its effect on yield (Zhao et al., 2017; Yao et al., 2014; Zhang et al., 2024). Previous studies have established a series of relationship models between NNI, AND, and RY based on differences in nitrogen uptake and utilization of different crops or crops at different growth stages (Zhao et al., 2022; Wang et al., 2010) however, obvious differences exist in nitrogen uptake, distribution, and utilization of different crops or crops at different growth stages. Therefore, the applicability and accuracy of this model must be further verified (Zhao et al., 2018). In this experiment, the relationship models of NNI, AND, and RY established based on maize RDM, SDM, LDM, and PDM reached a significant determination coefficient (*R* ^2^>0.80), which is consistent with the results of Su et al. (2021), indicating that the relationship models of NNI, AND, and RY showed high stability at different growth stages and in different maize organs. The model can be used to evaluate the nitrogen nutritional status of maize; however, there are obvious differences between organs and varieties. The response of maize roots to NNI was strong in the early growth stage and AND gradually weakened in later growth stages; whereas, the response to AND was weak in the early growth stage and gradually enhanced in the later growth stage, which is consistent with the results of Guo et al. (2024) and Wang et al. (2011) Nitrogen absorbed by roots in the early growth stage was mainly used for root morphogenesis and maintenance of its physiological function; whereas, in the later growth stage, root nitrogen was rapidly transferred to the kernel, and the nitrogen content decreased rapidly. The response of yield to root NNI was weakened and the response to AND was enhanced (Ata et al., 2014; Peng et al., 2009; Chun et al., 2005; Gao et al., 2023). In the early growth stage, the responses of the stem-sheath to NNI and AND were weak and gradually increased in the later growth stage, which is consistent with the results of Wang et al. (2010) In the early growth stages, plants preferentially allocate nitrogen to the roots and leaves to promote establishment of absorbing and photosynthetic organs. As an organ for nutrient and substance transport, the nitrogen status of the stem-sheath directly affects the grains. The response of leaves to NNI was strong in all stages; whereas, the response to AND was weak in the early stage and gradually increased in the later stage, which is consistent with the results of Su et al. (2021). As the most important photosynthetic organ of plants, plants preferentially distribute nitrogen to leaves during the vegetative growth stage, which not only contributes to chlorophyll synthesis but also improves photosynthetic efficiency. It can also significantly improve dry matter accumulation (Bian et al., 2019).

Further analysis of the relationship model of NNI, AND, and RY showed that ZH 311 roots, stem-sheath, leaves, and plants with high nitrogen efficiency were less responsive to AND than those of ZH 311 with low nitrogen efficiency at each growth stage; whereas, ZH 311 roots had a stronger response to NNI at the early growth stage, and stem-sheath, leaves, and plants had a stronger response to NNI at the later growth stage. The results showed that nitrogen-efficient varieties were not sensitive to nitrogen deficiency and were more efficient in nitrogen nutrition, which is consistent with the results of Qu et al. (2016) Nitrogen-efficient varieties have superior root structures, higher root biomass, and increased nitrogen uptake (Liu et al., 2019, Liu et al., 2022). Furthermore, these varieties have a stronger nitrogen transport capacity in the stem-sheath and leaves, which promotes efficient nitrogen use and results in each organ having a higher nitrogen concentration, which is insensitive to nitrogen deficiency (Hao et al., 2011; Du et al., 2024), has more efficient nitrogen nutrition, and can maintain higher dry matter accumulation and yield under nitrogen deficient conditions. We also found that under the same nitrogen concentration condition, the nitrogen dry matter production capacities (NDMP) of stem-sheath, leaves, and plants in different growth stages of the nitrogen-efficient variety ZH 311 were significantly higher than those of nitrogen-inefficient variety XY 508, which was consistent with the results of Ji et al (2014) in a study on rice. From another perspective, this study proved that the nitrogen efficient varieties were insensitive to nitrogen deficiency and efficient use of nitrogen nutrition.

## 5 Conclusion

In this study, critical nitrogen concentration dilution curves were established based on different organs of maize varieties with different nitrogen efficiencies, and the coefficients of determination (*R* ^2^>0.90) reached significance, which could be used for diagnosing maize nitrogen nutrition. The critical nitrogen dilution curves of the different organs of maize varieties with different nitrogen efficiencies differed significantly. The critical nitrogen concentration dilution model of each organ of the maize variety with high nitrogen efficiency, ZH 311, had a higher value than that of the maize variety with low nitrogen efficiency, XY 508, which was conducive to maintaining a higher nitrogen concentration in each organ during the later growth period and delayed aging of each organ. A lower b value (except for the roots) can slow the nitrogen dilution rate and maintain the nitrogen concentration in each organ. Further analysis showed that the nitrogen-efficient variety ZH 311 had the highest stability based on the stem-sheath, and XY 508 had the highest stability based on the leaves. RY, NNI, and AND were significantly correlated at different growth stages of different organs (*R* ^2^>0.80) using each critical nitrogen concentration dilution curve to predict yield. ZH 311 roots, stem-sheath, leaves, and plants with high nitrogen efficiency showed weaker responses to AND than those of ZH 311 with low nitrogen efficiency at all growth stages, and ZH 311 roots had stronger responses to NNI at the early growth stage, and the stem-sheath, leaves, and plants had stronger responses to NNI at the later growth stage, indicating that ZH 311 with high nitrogen efficiency was not sensitive to nitrogen deficiency and was more efficient for nitrogen nutrition. However, the research is limited by the singleness of varieties, the lack of analysis of environmental interaction and the lack of physiological mechanism. In the future, it is necessary to expand the universality of genetic and environmental diversity verification models and develop precise nitrogen diagnosis tools based on organ CNDCs to optimize maize nitrogen management strategies.

## Data Availability

The original contributions presented in the study are included in the article/supplementary material. Further inquiries can be directed to the corresponding authors.
